# Work context and drinking behavior in the French public service: a qualitative study

**DOI:** 10.3389/fpubh.2024.1432324

**Published:** 2024-10-28

**Authors:** Benjamin du Sartz de Vigneulles, Florence Carrouel, Guillaume Roucoux, Christian Michel, Catherine Massoubre, Geneviève Motyka, Elise Verot, Claude Dussart

**Affiliations:** ^1^Laboratory “Health, Systemic, Process” (P2S), UR4129, University Claude Bernard Lyon 1, University of Lyon, Lyon, France; ^2^Independent researcher, Paris, France; ^3^ECEVE, UMR-S, Inserm, Paris Cité University, Paris, France; ^4^Practice for Addiction Medicine - Association for Prevention and Rehabilitation (gGmbH), Kehl, Germany; ^5^Research Unit EA7423, Department of Psychiatry, Saint-Etienne University Hospital Center of Saint Etienne, University Jean Monnet, Saint-Étienne, France; ^6^Caisse Nationale d’Assurance Maladie, Paris, France; ^7^Institut Universitaire de Recherche PRESAGE, University Jean Monnet, Saint-Étienne, France; ^8^Equipe PREDUCAN, CIC Inserm 1408, Saint-Étienne, France; ^9^Hospices Civils de Lyon, Lyon, France

**Keywords:** alcohol, addiction, prevention, workplace, behavior, health promotion, public service

## Abstract

**Introduction:**

Alcohol use disorders (AUD) are a major public health problem. Among the working population, alcohol is the most frequently used psychoactive substance, as well as the most inappropriately used. Alcohol consumption reduces the worker’s psychophysical integrity, leads to an increase in inappropriate behavior, accidents and injuries, and affects the safety and health of other workers. Thus, the workplace must play an essential role in prevention. Particularly in France, in the civil service, a specific professional sector made up of many professionals exposed to stress, the prevention of AUD must play an essential role. The objective of this study was to describe the framework of alcohol consumption in the French public service in order to understand the sources of alcohol consumption behaviors and to identify the prevention measures to be implemented, in order to reduce the risk of transition to an alcohol use disorder.

**Methods:**

This descriptive qualitative study was based on focus groups. Sampling was purposive and data saturation was verified. Coding was “*in vivo*,” descriptive and pattern-oriented. Analysis was inductive thematic, and the COREQ guidelines were followed.

**Results:**

Firstly, the presence of alcohol in the workplace has been characterized by a before-and-after a prohibition law, and by the revelation of mechanisms for avoiding the ban. Then, the three main determinants for alcohol use disorders were: society, work and personal factors. Lastly, the prevention initiatives identified must be based on both collective and individual approaches. They must be encouraged by the hierarchy, include screening, accompaniment to care, and take into account both work in the workplace and work at home. The fight against work-related alcohol use disorders must be part of the corporate culture.

**Discussion:**

Preventing alcohol-related disorders among civil servants will help fight the burden of non-communicable diseases.

## 1 Introduction

Alcohol use disorders (AUD) are a major public health problem responsible for 3 million deaths every year (1). The consumption of alcohol, a psychoactive substance with addictive and toxic properties, is associated with increased risk of non-communicable diseases, infectious diseases and injuries (1, 2). It accounts for 5.1% of the global burden of morbidity and mortality (1). Mortality associated with alcohol consumption is greater than for diseases such as HIV/AIDS, tuberculosis, and diabetes (1). Among people aged 20 to 39, 13.5% of deaths are attributable to alcohol (1). Despite a 5% reduction in the number of drinkers worldwide, alcohol is still consumed by more than half the population in three WHO regions – the Americas, Europe and Western Pacific (1). In Europe, alcohol is responsible for around 800 deaths a day [cancer (29%), cirrhosis of the liver (20%), cardiovascular disease (19%) and injuries (18%)]. Alcohol impacts not only consumers, but also their families and the community, through the degradation of personal and professional relationships, criminal behavior, decreased productivity and healthcare costs (2, 3). In France, alcohol-related disorders affect around 7% of adults (3.5 million people) and were responsible for 41,000 deaths in 2015 (4). Among people with alcohol use disorders (AUD), less than half have sought mental health care in the past 12 months and approximately 10% have received related medical care (5).

A relationship between alcohol and work has been underlined in several studies (6–8). In addition to the negative influence on productivity and performance at work, the consumption of alcohol is associated with increased unemployment, reduced employment, absenteeism and the risk of accidents (2, 6). Among the working population, alcohol is the most frequently used psychoactive substance, as well as the most inappropriately used (9). Out of every 10 employees, 1–3 can be considered at-risk drinkers needing attention (10, 11), i.e., whose drinking habits lead to increased risk of medical, domestic, social, legal, occupational and economic problems (10).

In Europe in 2019, 8.4% of the adult working-age population drank alcohol daily, 28.8% weekly, 22.8% monthly and 26.2% never or not at all in the last 12 months (12). In addition, alcohol consumption is responsible for serious problems in 5–20% of this working population (13). Alcohol consumption reduces the worker’s psychophysical integrity, leads to an increase in inappropriate behavior, accidents and injuries, and affects the safety and health of other workers (2). To be more concise, in occupational sectors with a high risk of accidents (police forces, transport, etc.), workers who are under the effect of alcohol represent a danger to other workers (2, 14).

The workplace represents a living environment bringing together structural and social determinants of health (15) including alcohol consumption. Thus, the workplace influences workers via: (i) workplace alcohol beliefs (presence of alcohol, ease of drinking during breaks or work); (ii) descriptive norms (members of the worker’s social network work under the influence of alcohol or while consuming alcohol); and (iii) injunctive norms (members of the worker’s social network approve of working under the influence of alcohol or drinking while working) (16). The workplace can therefore be correlated with alcohol consumption and constitutes an opportunity to change risky behaviors according to personal motivation and individual abilities (17). Thus, the workplace must play an essential role in prevention (18).

In France, representations, beliefs and patterns of alcohol consumption make it difficult to implement effective prevention (19). Alcohol consumption represents a social and cultural norm from adolescence (20) and alcohol consumption is an element of daily social interactions (19). For example, wine in France or rum in the French West Indies, due to the history of their production and the tradition of their consumption, retains a positive image and leads to minimizing the damage that its consumption causes (21, 22). Furthermore, alcohol is consumed throughout the week unlike in certain other European countries where alcohol is mainly consumed on weekends (23). Additionally, as a large number of people work in the alcohol industry in France, the French government finds it difficult to completely discredit this drug (24). Unlike other countries like in the United Kingdom, for example, there are currently no plans to increase taxes on alcohol (19).

However, several occupational health and safety measures have been implemented to address the risks associated with alcohol consumption in the workplace. These measures are regulated under the Labor Code, which includes specific provisions such as articles R4228-20 (25) and R4228-21 (26) that limit the consumption of alcohol in professional settings. These regulations prohibit the presence of alcoholic beverage, except for wine, cider, beer or perry, in company restaurants or during special events like farewell evening or end-of-year party. Additionally, employers are encouraged to implement workplace health programs focusing on psychoactive substance use, including alcohol. Recently, initiatives such as “Dry January,” a public health campaign encouraging alcohol abstinence throughout the month of January (27), has been implemented in France since 2020 (28). As AUD represents a health burden, the French government has initiated political reflection on the use of psychoactive substances, including in the workplace (29, 30).

Despite these initiatives, comprehensive prevention strategies in the workplace, especially in the public service, remain underdeveloped. While some policies such as the National Health and Safety at Work Strategy (2016–2020) offer general guidelines on preventing alcohol-related harm, public service employees have historically lacked targeted prevention measures (31). The first national occupational health plan for the civil service, introduced in 2022, aims to address mental health and substance use disorders, encouraging the implementation of preventive measures such as employee assistance programs, stress management workshops, and stricter alcohol screening policies (32). However, the shortage of occupational health physicians and the lack of routine prevention programs tailored specifically for public service employees continue to pose significant barriers to effective prevention efforts.

Thus, the study of alcohol consumption in the specific socio-professional context of the French public service is interesting for several reasons. First, these public agents operate in a specific work context, with a job guarantee and relatively strong stability in their professional environment. Second, several socio-professional categories are represented (33). Third, several public service professions can be related to stress and psychological suffering, such as police officers (34), prison guards (35), or custom officers (36). Finally, this population works in a particular context with little prevention, since the first national occupational health plan for the civil service is recent (32) and there are few occupational health doctors in post.

The objective of this study was to describe the framework of alcohol consumption in the French public service in order to understand the sources of alcohol consumption behaviors and to identify the prevention measures to be implemented, in order to reduce the risk of transition to an alcohol use disorder.

## 2 Methods

This qualitative study followed the research protocol previously published (37) and was carried out in the public service workplace in France. The choice to conduct qualitative studies is based on the potential that this type of research can contribute to understanding a problem in various dimensions or to study phenomena not yet detected (38). It focused on a descriptive design based on the framework of alcohol use in the public service workplace.

This research was performed in accordance with the Consolidated criteria for reporting qualitative research (COREQ) guidelines (39) (Supplementary File 1).

### 2.1 Sample recruitment

The sampling method was purposive (40). Participants received an email containing the recruitment announcement, the study information sheet (with ethics approval references), and the consent document. Participants gave written informed consent for the focus groups to be recorded, full verbatim transcribed and the data being published anonymously.

### 2.2 Inclusion and exclusion criteria

Inclusion criteria were: (i) over 18 years old, (ii) active or retired civil servants, (iii) mutualist activists and, (iv) representatives of the Local Health Insurance Section (SLAM, Section Locale d’Assurance Maladie) responsible for implementing preventive actions in public administrations on behalf of the Union of Health Prevention for the Obligatory System (*Union prévention santé pour la Fonction publique, Urops*).

Exclusion criteria were: (i) lack of consent form; (ii) inability to participate in the focus group; and (iii) early departure during the focus group.

### 2.3 Interview guide

The interview guide was composed of three parts (37). The first part (nine open-ended questions) focused on the topic of alcohol and work to analyze the link between alcohol consumption and the professional social framework, typologies of alcohol consumption at workplace, perceived risks, and specificities in the public service. The second part (three open-ended questions) focused on the topic of alcohol and psychological suffering to explore the link between alcohol consumption and mental health, the mental state of civil servants and its impact on alcohol consumption. The third part (one question) focused on solutions.

The interview guide was subsequently presented to two SLAM representatives, out of focus groups, to confirm its relevance to the target audience. The same interview guide structure was used for both studies.

The form and content of the interview guide were validated by a PhD health professional, specialized in the addiction research field (CM). The same guide was used for all focus groups.

### 2.4 Data collection

Data collection was carried out through five focus groups held in France between November 2022 and January 2023.

To conduct the focus group, two men acted as animators. CM has a PhD and is a physician specializing in addictology. BD has a MSc and is a doctoral student in public health. BD was the main animator who steered the discussions according to the interview guide, and CM acted as an observer who ensured that the research ran as planned, and could intervene if necessary. These two researchers were trained in qualitative studies and had no prior relationship with participants. Participants were informed that the goal of this research was to determine the preventive measures against AUD.

The framework in which the collection took place was explained to each group of participants, ensuring their freedom of speech, data protection, anonymisation, and policy of non-reporting to their hierarchy.

The focus group took place in meeting rooms outside the participants’ place of work and in the absence of non-participants. Each participant realized one focus group interview which was recorded without taking notes. Discussions were transcribed in verbatim form by a qualified researcher. The moderators checked the accuracy of the transcripts. Feedback regarding focus groups was asked to the participants.

### 2.5 Qualitative data analysis

The five steps methodology framework proposed by Braun and Clarke to conduct the thematic analysis were applied (41). Nvivo 14 software (QSR International) was used to perform the analyses.

1. Familiarization with the data. This phase involves reading and rereading the data, to become immersed and intimately familiar with its content (BD, PhDc; FC, PhD and GR, PhDc).
2. Generating initial Codes: this phase involves generating succinct codes that identify important features of the data that might be relevant to answering our research question. It involves coding the entire dataset, and after that, collating all the codes and all relevant data extracts, together for later stages of analysis (BD, PhDc; FC, PhD and GR, PhDc).
3. Searching for themes: the collection of codes was worked on by the three researchers (BD, PhDc; FC, PhD and GR, PhDc) to validate a comprehensive interpretation and a grouping of code elements into themes.
4. The resulting themes were then compared and discussed between the researchers.
5. Finally, a more refined coding was used for each part of the verbatim used to illustrate the themes.

### 2.6 Ethical considerations

The study was conducted in accordance with the Declaration of Helsinki, and approved by the Research Ethics Committee of the University of Lyon (n°2022-09-15-004 on 18 October 2022). All participants were given written and oral information about the study and gave informed consent to participate.

## 3 Results

### 3.1 Characteristics of focus group participants

Among the 75 SLAM representatives who received the email presenting the study, 30 accepted to participate (40%) and were divided into five focus groups. The participants represented a wide variety of ages, seniority and experience as described in Table 1. Group size ranged from four to eight with an average of six participants. Sixty percent of participants (*n* = 18) were male, 53.33% (*n* = 16) were retired and 70.00% were over 60 years old. Regarding the public service categories, 30.00% (*n* = 9) was from category A (conceptual, managerial and supervisory grades and positions, as well as teaching positions), 40.00% (*n* = 12) from category B (middle management, application and editorial positions) and 30.00% (*n* = 9) was from category C (executive positions). In terms of length of service in the public sector, 93.33% (*n* = 28) had more than 10 years’ seniority.

**Table 1:** Characteristics of the 30 participants.

Variables	*N* (%)
Gender	
Man	18 (60.00)
Woman	12 (40.00)
Profession status	
Active	16 (53.33)
Retired	14 (46.67)
Age groups	
30–44 years	2 (6.67)
45–59 years	7 (23.22)
≥ 60 years	21 (70.00)
Public service category^1^	
A	9 (30.00)
B	12 (40.00)
C	9 (30.00)
Public service seniority	
5–10 years	2 (6.67)
> 10 years	28 (93.33)
Seniority as SLAM representative	
< 5 years	14 (46.67)
5–10 years	9 (30.00)
> 10 years	7 (23.33)

^1The French public service distinguishes three main categories of workers: Category A covers hierarchically superior conceptual, managerial and supervisory grades and positions, as well as teaching positions, Category B concerns middle management, application and editorial positions, Category C includes executive positions. The conditions of access to these categories vary according to the level of qualification and graduation. The basic remuneration is different between these categories. There is also the category of “contractual” or “non-permanent”.^

Debriefing session followed each focus group discussion. All participants were enthusiastic about speaking out on the subject of alcohol and its uses in their professional environment.

The average duration of each focus group was 1 h46 (min 1 h35–max 2 h03). They were grouped by topic. A total of 48 topics were discussed in the first focus group. Seven other topics were discussed in the second focus group. Then, again, seven other topics were discussed in the third focus group. The fourth and fifth focus groups did not reveal any additional topics, confirming data saturation and the analysis reliability. Thus, 62 topics were covered.

### 3.2 Major themes and sub themes emerging

The qualitative analysis revealed three main themes, each associated with three sub-themes. Table 2 provides an overview of the categorization:

- The presence of alcohol consumption in the public service
- The sources of alcohol consumption behaviors
- The appropriate prevention actions

**Table 2:** Overview of major themes and subthemes associated.

Major theme	Presence of alcohol consumption in the public service	Sources of alcohol consumption behaviors in the public service	Appropriate prevention actions
Sub theme	In the past, excess of alcohol Nowadays, the ban in the workplace and its consequences Deviations from today’s ban	In society The work The personal factors	Collectives approaches Individual approaches Doing nothing

#### 3.2.1 The presence of alcohol consumption in the public service

The first content theme (illustrated by 459 verbatims) to emerge was the presence of alcohol consumption in the public service. Three sub-themes were developed (Table 2): (i) in the past, excess of alcohol, (ii) nowadays, the ban in the workplace and its consequences, (iii) deviations from today’s ban.

- In the past, excess of alcohol:

In the past, alcohol consumption at work in the public service was habitual, frequent and high. The opportunities for drinking were many and varied. The alcohol consumption was considered as necessary:

*“Every day, another reason to drink something” (n°12).*

Access to alcohol in the workplace was easy. The possession of alcohol by civil servants in their offices was commonplace and was facilitated by the structure itself:

*“I’ve known departments where there were people who had their drawer, and their bottle right in the drawer, and from time to time, there you go” (n°42).*

Alcohol-related disorders could affect everyone, and all socio-professional categories. It is in this sense that it has been observed that social position is waning in the face of alcohol. People in positions of authority (chiefs, directors) could also be alcoholized at work:

*“Managers like any of us and even outside the public service are people, so they have problems and have different reactions, some like the rest of the population being the use of alcohol as an escape or as a way of forgetting “(n°26).*

In addition, alcohol could be perceived as a symbol of performance or shared culture:

*“we had to have the strength, the ability, to overtake those who could overtake us, by the product itself [implied alcohol], and show that we were able to endure” (n°51).*

Furthermore, alcohol was associated with moments of conviviality, and seen as a facilitator of working relationships:

*“Alcohol was the aperitif that allowed us to better discuss” (n°53).*

It seems that professional traditions could have been conducive to unlimited alcohol consumption.

*“There are traditions every day. In the Army, there are lots of traditions. Before I came [here to the focus group], there was Sainte Barbe, there was Saint Eloi, there’s always… and now I’m back on Wednesday, Friday there will be the Colonial breakfast” (n°24).*

Accident associated with alcohol consumption was not considered by the professional structure:

*“There’s one thing that’s been really important. It’s the responsibility of agents, particularly in terms of driving. That is to say, in the old days, before the 85/90s perhaps, a civil servant who had a serious car accident,* etc.*, due to alchoholism, posed no problem for the administration. I remember some memos from around 2000, which said: “Now, agents are responsible, and all the more so if it’s a company vehicle” (n°51).*

- Nowadays, the ban in the workplace and its consequences:

Nowadays, it appears that the reduction in alcohol consumption in the workplace is due to the introduction of a law that has led to material and practical changes, such as alcohol-free social events or even their disappearance.

*“And now, it is true that for a few years, for quite a few years now, with the regulations, it is true that there are almost, there are no more convivial drink moments” (n°42).*

These changes can have psychosocial impacts and consequences on mentalities and behaviors:

*“And this frustration [of not having alcohol at work, and going somewhere else] bothers me” (n°27).*

We can also notice that alcohol has become a taboo subject:

*“No, but what I meant was that alcohol at work is not talked about” (n°24).*

Indeed, compliance with the ban on alcohol in the workplace is verified, offices can be inspected, and breaches can be sanctioned:

*“there’s the inspector who comes in, checking the offices to make sure we do not have cupboards with bottles or things like that” (n°55).*

The prevalence of alcohol consumption and the agents concerned by alcohol use disorders in the workplace may appear to be declining:

*“So over the last few years, it’s calmed down a little bit everywhere” (n°12).*

This decline in prevalence seems to be accompanied by a reduction in social pressure to drink alcohol:

*“Now, it’s not heard anymore. If you do not feel like drinking alcohol, even cider, you drink orange juice, and that’s fine. There’s no longer that stigma” (n°42).*

However, the ban on alcohol in the workplace can be circumvented by implementing avoidance strategies. People drink outside the workplace, for example, lunch outside is an opportunity to consume alcohol:

*“And the alternative, cheating, that’s done [alcohol consumption] elsewhere” (n°26).*

- Deviations from today’s ban

However, there seems to be a persistent presence of alcohol in the workplace that can indirect or direct:

*“People who go out for lunch, who have a punch [drink with rum], who have wine, who have beer off the work site, but who come back with alcohol in their blood, they are drunk” (n°24).*

The ban can be differentially interpreted and the presence of alcohol in the workplace remains tolerated:

*“it’s just… then everyone interprets it the way they want” (n°24).*

It turns out that cases of drunkenness persist in the workplace:

*“Now, we have both, we can have… we can experience two situations: people who are addicted to alcohol, and then others who are hyper athletes and rarely have a drink” (n°14).*

The relevance of alcohol prohibition in the workplace is questioned in cases where work is not affected:

*“as long as it does not affect his work, if it does not affect the department’s activity, it’s hard to see why he should be sanctioned” (n°34).*

Also, there may be a shift to private space, with the development of teleworking, work from home:

*“Now, that makes me wonder. It raises the issue of working from home and alcohol. Because the law regulates alcohol at work, but what is it when you work from home?” (n°44).*

#### 3.2.2 The sources of alcohol consumption behaviors in the public service

The second content theme (illustrated by 239 verbatims) to emerge was the sources of alcohol consumption behaviors in the public service. Three different content codes were developed: (i) in society, (ii) the work, (iii) personal factors (Table 2).

- In society:

Alcohol consumption is omnipresent, and can be found in all sectors of professional activity and at all socio-professional levels. And the disorders that can be associated with it have multiple origins.

*“There are people who have major problems with alcohol in all the… I do not think it’s specific to one public administration or another […] The origin can be multiple” (n°35).*

Alcohol is a cultural product, a form of heritage, liable to bring pride:

*“Everyone wanted me to bring back X [name of a brand of local alcohol], even though it is a product that is everywhere. That’s the proof that it’s cultural” (n°56).*

Alcohol can be seen as an element of conviviality and sharing, of cohesion and socialization:

*“it was alcohol socialization. In other words, we socialize through alcohol” (n°56).*

But these social relationships organized around alcohol can lead to higher levels of consumption that can initiate the addiction phenomenon.

*“But then, when we are several? well, there’s someone who buys his round of booze, the second, the third and then it’s a chain after that” (n°31).*

And then there are specific social circumstances that can encourage alcohol consumption:

*“There was Covid too, we must not forget that. It’s not just telecommuting, it’s Covid too, which has generated additional stress. In fact, as we have seen, mental health problems have increased significantly” (n°53).*

- The work:

Work can be seen as an emotional destabilizer, and the nature of the work can be a determining factor in alcohol consumption:

*“But I know some too, it’s still… it’s work what, it’s work that makes them drink” (n°13).*

Pressure to perform professional tasks, the pace of work and difficult working conditions are seen as factors that can encourage alcohol consumption. Work can lead to psychological fatigue of employees, a feeling of ill-being or even a burn-out:

*“But the guy, because he has an ill-being at work, drinks because of it” (n°13).*

Working conditions can also have an impact on ancillary sources of emotional stability, such as family separation for professional reasons, as in the police force for example:

*“the difficult context of [professional] travels and the social situation that is not easy for colleagues to live with, leads to decompression by alcohol” (n°14).*

But also, absence of work can be a cause of alcohol consumption. For civil servants, retirement is the main cause of absence of work. And this state is seen as a possible source of alcohol consumption:

*“About retirement, I knew an officer who worked, he was a police officer, I’m not going to name him. And he came in every morning - I met him at the end of his career - and he came with fear in his stomach […], then he started drinking [in retirement] and in fact, he ended up with… he committed suicide […] All his frustrations, well, they came out” (n°33).*

Periods of confinement during the COVID health crisis or the loss of a job are also situations that can lead to increased alcohol consumption:

*“Because someone who does not have a reason to keep busy, well that’s catastrophic [regarding alcohol consumption]” (n°34).*

- Personal factors:

Two endogenous characteristics are mainly invoked in relation to alcohol consumption and use disorders. Firstly, individual genetic characteristics:

*“Sometimes, there’s already something genetic, and then that triggers it as well. But in my opinion, there is something. Well, I’m not a scientist, but I think that genetics… from generation to generation, sometimes it comes out” (n°12).*

Second, individual psychic characteristics, related to a weakness of character or personality:

*“I find that we encourage weak people to drink [social pressure to drink alcohol during social occasions at work], rather than protecting them” (n°31).*

Early exposure to alcohol, or even education in alcohol consumption, is cited as a source of personal factors that may explain the transition to a substance use disorder:

*“since we were little, we had this pattern of alcohol. So afterwards, you take your punch…” (n°23).*

Vulnerability factors in personal life, family problems and financial problems can also be a source of behavior leading to drinking disorders:

*“And many civil servants, when there are personal worries, turn to alcohol” (n°33).*

Alcohol can be seen as a marker of fragility in social integration:

*“And we can see, without it being pejorative, but the ‘social cases’ [people in precarious situations], without it being pejorative, I repeat, all the people who have really had a lot of problems with alcohol in particular” (n°56).*

Lastly, it results that alcohol can then be considered as a help in the face of ill-being, stress, pressure, annoyance, fatigue, worries, mental or physical pain:

*“we could say that workloads are changing, and that this is causing more stress, perhaps for agents, and that they are trying to find reasons, ultimately, to decompensate by consuming alcohol” (n°53).*

*“mental health problems have increased significantly because of Covid, and that may also explain the increase in consumption” (n°53).*

*“Also, when you are very tired, a little shot of alcohol, it… (gesture mimicking tonus) A little one” (n°58).*

*“Some people think that by drinking alcohol, it can at least relieve some pains” (n°25).*

#### 3.2.3 The appropriate prevention actions

**Figure 1:**
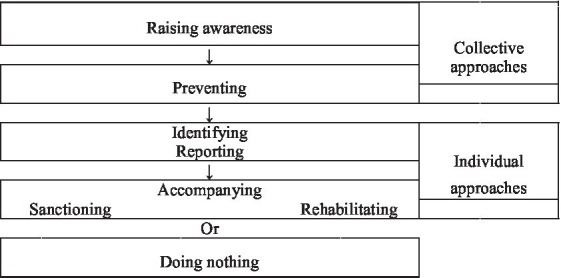
Appropriate prevention actions against alcohol consumption disorders in the public service.

The third content theme (illustrated by 708 verbatims) to emerge was about prevention actions to reduce the risk of transition to an alcohol use disorder. Three different content codes were developed: (i) collective approaches, (ii) individual approaches, or (iii) doing nothing (Figure 1).

- Collective approaches

Raising awareness of AUD must be done in the context of addictions in general in order to attract as many people as possible because this topic is different from others dealing with health risk factors:

*“because if we’d only done alcohol addiction, I cannot even imagine, we’d have had even fewer people […] Do a prevention campaign just by saying “addictions,” no one comes” (n°42).*

Some participants felt that prevention actions should be voluntary and open to everyone:

*“After that, it is true that the population we touched with the Addictoquiz (name of a serious game), it was not necessarily people who were addicted to something. Everyone signs up, no matter” (n°55).*

However, other participants indicated that it is necessary to gain access to people and that, therefore the obligation to participate in actions may have advantages:

*“They did three conferences, but you had an obligation to go […] And in the mountains, it was the brigade director who forced the officers to go” (n°56).*

Preventive actions must include education, communication with clear messages, be entertaining and be long-term:

*“Yes [education], it’s a minimum” (n°37).*

The alcohol use topic should be included in more general actions such as the promotion of well-being or health and be adapted to the individual:

*“It should perhaps be integrated [the alcohol topic] into a well-being action […]. For example, if someone like me likes sport, we could say to them ~ ‘listen, you like sport, but alcohol is not good for your performance’“(n°44).*

These preventive measures must also take the form of measures that can use prohibitions and/or obligations to standardize behavior:

*“It’s a bit the same. If there was a breathalyzer at the entrance to buildings, and people had to blow out their breath systematically, just like badging. I think there would be some grumbling at first, but after that it would quickly become standard practice” (n°44).*

But also through participative actions, because the motivation to commit to changes in health behaviors can be strengthened by collective stimulation.

*“There is a group effect [in months without alcohol]. People need that” (n°43).*

The preventive approach must be part of a clear political structure with committed directions. And to encourage management commitment, financial remuneration is mentioned:

*“there would already be a common policy on the apprehension of alcohol in all departments, throughout the public service” (n°37).*

However, prevention requires human, financial and technical resources:

*“I think it’s the resources. You need the resources” (n°33).*

- Individual approaches

The importance of the identification of persons suffering from AUD was underlined. For some, AUD are easily detectable:

*“everyone can see it” (n°13).*

But these disorders can also be seen in the application to the professional task and the quality of the work done, or in morale and the quality of relationships:

*“Because the person who is going to become dependent is going to be worse and worse, quality at work, quality of family life too” (n°11).*

Identification should take place during interviews carried out as part of a professional activity, such as a medical examination:

*“I think it’s during the professional interview [where alcohol can be discussed]” (n°56).*

Targeted actions such as the use of breathalyzers could be used to identify people with AUD. However, the problem of accreditation of such checks, acceptability by civil servants or syndicats, the risk of positive tests and the cost is put forward.

*“The idea is very good [a car that will not start if the driver is drunk], but I’m going to have to worry about the means of doing it” (n°37).*

Participants underlined the importance of reporting cases of AUD but this is countered by the notions of denunciation and responsibility:

*“that’s it, you have to go and denounce” (no. 34).*

*“It’s not denunciation, it’s support” (n°32).*

Reporting also means delegating responsibility:

*“I report it to exonerate myself” (n°37).*

However, it appears that it is not easy to report a problem related to alcohol consumption, the error is possible, and it can sometimes be seen as a private matter:

*“So, I go there, I go to see my director, we knew each other well, she knew the guy very well and when I told her: “oh there?! No, no, we must say nothing, we must do nothing, we will not bother him” — O right? What can I do after that?” (n°33).*

The participants mentioned the need of accompanying people identified as having an AUD and thus, the need of referral person:

*“that’s why it’s important to have someone within the… within the structure who is… trained for it” (n°14).*

The participants pointed out that sometimes resources (occupational physician, social services) exist but with limited power to act:

*“but the social worker, even if she knows of a case, well she cannot say anything, she does not have the right” (n°13).*

In some cases, the civil servants responsible for implementing health and safety rules are also prevention assistants or advisors, like

“*Sentinels” and “ACMO”* (*Agent Chargé de la Mise en Œuvre des règles d’hygiène et de sécurité*, *Health and Safety Officer*) (n°44).

Empathy in the workplace and solidarity are mentioned:

*“You have a colleague next to you who is obviously drunk, so you do what a human being would do,* i.e.*, you suggest that they do not take their car” (n°35).*

Sanctions were not perceived as a solution, managers’ views on the matter changed, and individual’s rehabilitation seems to be sought through care:

*“And he [the colleague sanctioned for alcohol] is going to feel diminished […] and we will not necessarily solve the problem of alcohol” (n°14).*

*But “in any case, these guys, if you put them in a treatment center because they have done something stupid, because they drink, and if you do not have any real psychological follow-up, it’s useless” (n°56).*

- Doing nothing

But sometimes, nothing is done for the employee with alcohol consumption problems:

*“because that’s what we were talking about too, people who feel bad. I think I’ve got three friends like that, they had problems at work, they did not get any help” (n°27).*

*“I do not feel that the space for intervention in public administration has been codified in such a way as to make it easy to correct problems” (n°57).*

*“They tell me: “yes, we know, but we turn a blind eye” […] I know for a fact that there will not be anything […] The follow-up is a mess, it’s disjointed” (n°37).*

*“because our leaders, all they want is for them to have numbers that are in their ways […] I’m going to be even tougher, but our managers aren’t interested. Our managers aren’t interested” (n°24).*

*“But they avoid the problem” (n°12).*

It is however reminded that:

*“Hiding does not help, it only makes it worse and it’s not doing anyone any favors” (n°43).*

## 4 Discussion

This study describes the framework of alcohol consumption in the French public service in order to understand the sources of alcohol consumption behaviors and to identify the prevention measures to be implemented, in order to reduce the risk of transition to an alcohol use disorder.

First, regarding alcohol consumption in the workplace, the study showed that it had been significantly reduced by the introduction of a law prohibiting alcohol consumption in the workplace even if the ban is not always respected. Indeed in 2008, article R4228-21 of the French Labor Code prohibited the entry or presence of intoxicated persons in the workplace (26). In 2014, article R4228-20 of the Labor Code prohibits alcoholic beverages other than wine, beer, cider and perry in the workplace {Citation} These alcoholic beverages may be served in the company restaurant or at special events (e.g., farewell parties, end-of-year celebrations, etc.). Any company wishing to prohibit the presence of alcohol in the workplace can do so via its internal regulations. In 2022, article L4622-2 reinforces the role of the employer, who must organize the provision of services in such a way as to avoid any negative impact on workers’ health (42). The employer must prevent the consumption of alcohol in the workplace. However, the Labor Code only authorizes systematic screening for alcohol consumption among employees performing dangerous tasks. Thus, France, like 18 European union member states (Austria, Belgium, Bulgaria, Croatia, Cyprus, Czech Republic…), has adopted an approach where laws regulate the phenomenon and impose restrictions on alcohol consumption (2). Four other countries (Ireland, Luxembourg, Portugal and Sweden) have delegated the power to control alcohol consumption to the employer, while others (Greece, Malta, the Netherlands and Romania) have no specific rules.

Second, despite the fact that the law restricts alcohol consumption in the workplace, this study showed that alcohol is still present in the workplace in all sectors of professional activity and at all socio-professional levels. Alcohol may be associated not only with the workplace, but also with individual factors (genetic, psychological…), educational factors, stress factors (personal difficulties, financial problems, family problems…) and cultural factors. Thus, this study fits into the socio-ecological framework explaining influences on alcohol consumption described by Sudhinaraset et al. (43). According to this model, individual factors influencing alcohol consumption are linked to the microsystem (family, work and school environments), which in turn are linked to the wider community (community, cultural, gender norms…). Macroeconomic factors, such as exposure to advertising, can influence the attitudes and norms of the family and peer network, which ultimately influence individual attitudes and behavior. Although, alcohol has been shown to be a risk factor for many chronic diseases and conditions (44), this study has demonstrated, as previously described in other contexts (8, 45, 46) that in the workplace, alcohol remains considered as an element of conviviality and sharing, of cohesion and socialization. The main problem is that the social anchoring of alcohol is one step of the addiction phenomenon. In addition, alcohol is described as a source of relief that can help to fight against the stress that represents the work. Some working conditions (shift or night work, long hours, remote working, physical danger and interface with a demanding or aggressive public, etc.) are associated with stress and alcohol consumption (47). In France, the state public service that represents 44% of public-sector employment with 2,52 million of agents in 2021, which comprises almost 10% of police officers, customs officers and prison guards (48), has the majority of its workers in occupations associated with stress. They are also more exposed to family instability (separation, divorce…) due to their profession and working conditions (working hours, transfers…). Alcohol is thus perceived as a help. This maladaptive coping mechanism of alcohol consumption was analyzed in several studies (49–51). Furthermore, in this study, participants pointed out that certain public service professions (military…) could be retired quite young (as early as 52 years) and that this change, this reduction in activity could be associated with an increase in alcohol consumption. As a result, many civil servants appear to be at risk of alcohol-related disorders, and preventive measures are essential.

Third, the workplace appears as an opportunity for implementing prevention programs because the main part of adults are employed, full-time employees spend an important proportion of their time at the workplace and work plays an important role in most people’s lives (52–54). A key element of preventive programs is the identification of persons suffering from disorders. For this, the managers could use interviews carried out as part of a professional activity, but they need to be trained (53). Médical physiciens could also be further educated in the detection of AUD. Each worker can also play a role in detection, but while some equate this with solidarity, others equate it with denunciation. In addition, other detection options, such as alcohol testing, have been proposed. However, current French legislation does not allow this, except for certain high-risk professions (2). After this step of detection, it is important to accompany the worker suffering from these disorders. Consideration of alcohol screening and brief interventions by the Capability, Opportunity, Motivation and Behaviour model (COM-B) (17) has shown several advantages (55) and may be needed for successfully implementing preventive actions and intervention functions to strengthen health behaviors 9/24/24 9:54:00 PM. Moreover, prevention campaigns can be adapted to the different levels of alcohol health literacy (56, 57) of employees and employers, and monitored by an alcohol use disorders test (58). However, significant improvement in employees’ knowledge of alcohol does not necessarily translate into significant effects on alcohol consumption (59); maybe due to lack of risk perception (60) or denial of problem drinking (61). Thus, it would be important to regularly maintain motivation for appropriate health behavior over the long term. Work-related AUD are a broader prevention target with the development of telework from home. In 2022, 23% of the state public servants teleworked at least one day a week (62). The implementation of participative collective actions, which can be carried out at any time or any place, such as “Dry January” (63, 64), can facilitate changes in health behavior. The support of people suffering from AUD in the professional context must also be able to go toward care, without stigma, and in the benevolence of professional staff. Moreover, managing this burden must involve a cultural approach: due to a link between the use of psychoactive substances and ill-being, prevention of AUD must also involve the prevention of psychological suffering linked to the professional task. This can particularly apply to the public service, a specific work context which is at the origin of the collective identity, based on soliditarity. Indeed, with the development of new public management, essentially based on a budgetary pragmatism and austerity (65), some structural injustices at work were observed (66) and a lesser social consideration of this sector is sometimes denounced (67). In this study, alcohol is strongly associated with celebration, sharing, and socialization. Thus, the ban on alcohol in this workplace seems to be accompanied by a decline in professional conviviality and cooperation. This seems to reinforce the perception of a cultural shift toward an erosion of the sharing of values, which are the source of the signification of the activity and the motivation of the engagement (68). Interventions that involve in-depth cultural adaptation may be more likely to be effective (69). Health prevention must therefore become part of the corporate culture, with the commitment of the top management (53), and must be adapted to the specificities of the workplace (52). This highlights the necessity of designing prevention measures that are not only tailored to the workplace but are also planned for the medium and long term, ensuring sustained effectiveness. This is a major challenge, because preventing the health of civil servants involves issues that range from individual well-being to the general public interest.

This study has several limitations. First, the selection of participants may have led to a “confirmation bias,” as people tend to report information that is consistent with their beliefs and to interpret the information they do have in favor of their preferred hypotheses (70). Second, participants were mostly from the state public service. They did not cover the entire French public service: the national education sector and the hospital sector, for example, were not present. However, they covered a wide variety of professions. Finally, because of the qualitative approach used in this study, it is not possible to generalize these findings to a broader population. However, the sample size is compatible with reliable qualitative analysis (71).

## 5 Conclusion

Overall, this study allowed us to explore the perceptions and representations of representatives of the Local Health Insurance Section responsible for implementing preventive actions in public administrations on behalf of the Union of Health Prevention for the Obligatory System, about alcohol consumption in the French public service. Although they consider that the introduction of legislation regulating alcohol consumption in the workplace has been associated with a reduction in consumption in France even if alcohol is still present in the public service. Thus, preventive measures to combat AUD need to be implemented. These actions must be part of general health initiatives, and should aim to prevent consumption, identify those suffering from alcohol or other use disorders and provide support.

## Data Availability

The raw data supporting the conclusions of this article will be made available by the authors, without undue reservation.
