# Supplementary material for: Work context and drinking behavior in the French public service: a qualitative study
Source: Front Public Health. 2024 Oct 28;12:1432324. doi: 10.3389/fpubh.2024.1432324 (PMC11555563; doi:10.3389/fpubh.2024.1432324)
Supplement: Supplementary file 1 [file Table_1.DOCX]

Supplementary Material

**Supplementary file 1.** Consolidated criteria for reporting qualitative research (COREQ) checklist


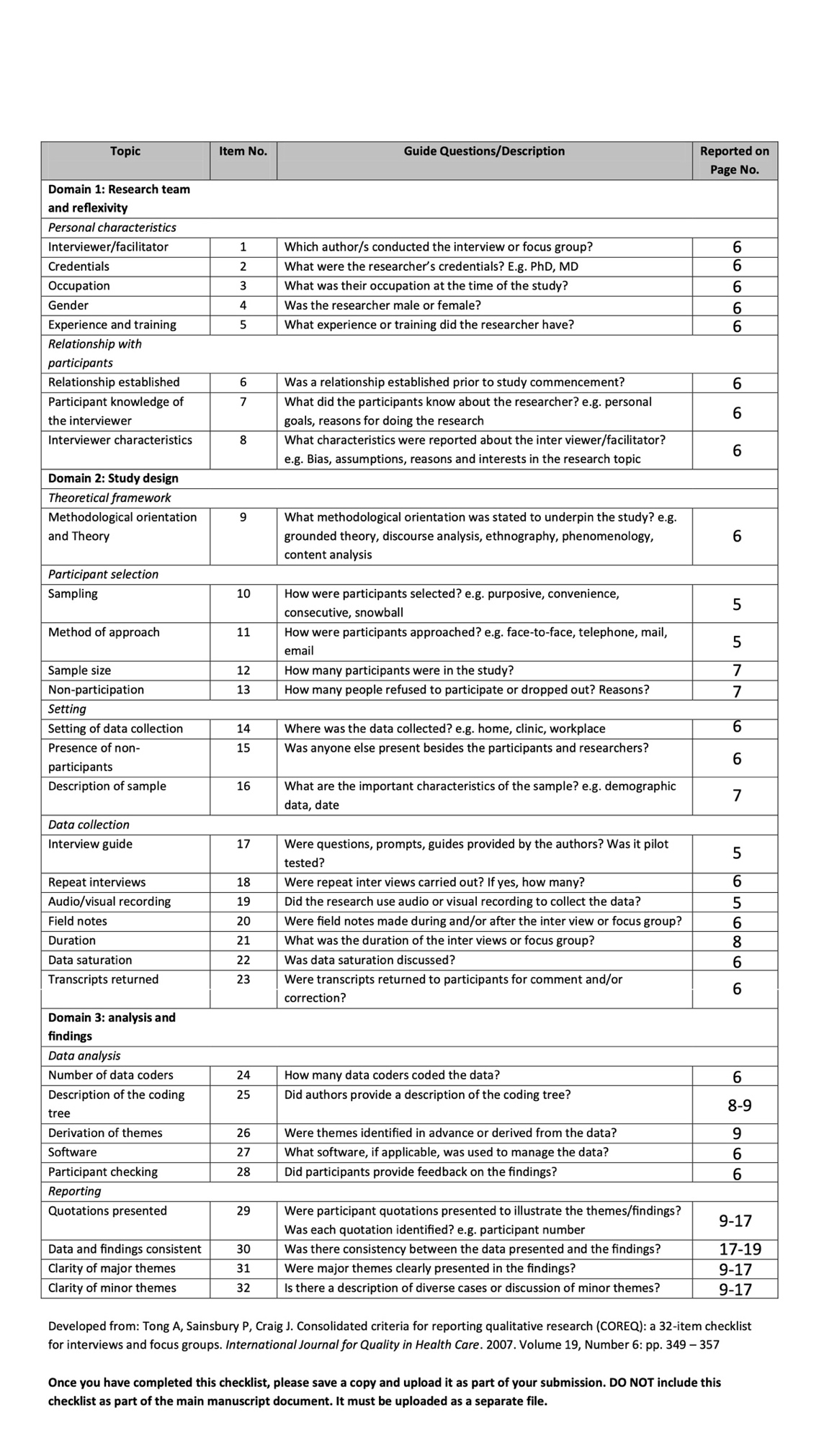


**Supplementary file 2.** Overview of major themes and subthemes associated with their characteristics

| **Themes** | **Sub-themes** | **Characteristics** |
| --- | --- | --- |
| Presence of alcohol consumption in the public service | In the past, excess of alcohol  (111 quotes) | High frequency  Easy access  Large quantities  Social position is waning in the face of alcohol  Symbol of performance or shared culture  Absence of limits |
|  | Today, the ban in the workplace and its consequences  (229 quotes) | Material changes  Changes in practices  Psychosocial impacts  Alcohol as taboo subject  Verifications and sanctions  Prevalence decline  Avoidance strategies |
|  | Deviations from today's ban  (119 quotes) | Persistent presence  Tolerance for the presence  Cases of drunkenness in the workplace  Questioning the relevance of the prohibition  Shift to private space |
| Sources of alcohol consumption behaviors in the public service | In society  (79 quotes) | Omnipresence and multiple origins  Cultural heritage  Socialization  Circumstances |
|  | The work  (68 quotes) | Nature of work  Work pressure  Impacts on ancillary stability  Absence of work |
|  | The personal factors  (89 quotes) | Endogenous characteristics  Early exposure and education  Vulnerability factors in personal life  Ill-being |
| Appropriate prevention actions | Collective approaches  (286 quotes) | Raising awareness  Preventing |
|  | Individual approaches  (365 quotes) | Identifying  Reporting  Accompanying  Sanctioning  Rehabilitating |
|  | Doing nothing  (47 quotes) |  |
